# Evaluating observer agreement of scoring systems for foot integrity and footrot lesions in sheep

**DOI:** 10.1186/1746-6148-8-65

**Published:** 2012-05-25

**Authors:** Alessandro Foddai, Laura E Green, Sam A Mason, Jasmeet Kaler

**Affiliations:** 1 Quantitative Veterinary Epidemiology group, Wageningen Institute of Animal Sciences, Wageningen University, Wageningen, The Netherlands; 2 School of Life Sciences, University of Warwick, Coventry, England, CV4 7AL, UK; 3 The School of Veterinary Medicine and Science, University of Nottingham, Sutton Bonington Campus, Sutton Bonington, Loughborough, Leicestershire England, LE12 5RD, UK

## Abstract

**Background:**

A scoring scale with five ordinal categories is used for visual diagnosis of footrot in sheep and to study its epidemiology and control. More recently a 4 point ordinal scale has been used by researchers to score foot integrity (wall and sole horn damage) in sheep. There is no information on observer agreement using either of these scales. Observer agreement for ordinal scores is usually estimated by single measure values such as weighted kappa or Kendall’s coefficient of concordance which provide no information where the disagreement lies. Modeling techniques such as latent class models provide information on both observer bias and whether observers have different thresholds at which they change the score given. In this paper we use weighted kappa and located latent class modeling to explore observer agreement when scoring footrot lesions (using photographs and videos) and foot integrity (using post mortem specimens) in sheep. We used 3 observers and 80 photographs and videos and 80 feet respectively.

**Results:**

Both footrot and foot integrity scoring scales were more consistent within observers than between. The weighted kappa values between observers for both footrot and integrity scoring scales ranged from moderate to substantial. There was disagreement between observers with both observer bias and different thresholds between score values. The between observer thresholds were different for scores 1 and 2 for footrot (using photographs and videos) and for all scores for integrity (both walls and soles). The within observer agreement was higher with weighted kappa values ranging from substantial to almost perfect. Within observer thresholds were also more consistent than between observer thresholds. Scoring using photographs was less variable than scoring using video clips or feet.

**Conclusions:**

Latent class modeling is a useful method for exploring components of disagreement within and between observers and this information could be used when developing a scoring system to improve reliability.

## Background

In the UK in 2004, 97% of farmers reported lame sheep in their flock with an average within flock prevalence of 10% [1]. Footrot is the main cause of lameness and foot lesions in sheep in the UK [2]. *Dichelobacter nodosus* is the essential organism for causing footrot, other organisms especially *Fusobacterium necrophorum* are thought to play an important role in the pathogenesis of footrot [3,4]. The clinical presentation of footrot is highly variable and ranges from mild interdigital inflammation (benign footrot) to under-running of horn with a characteristic smell (virulent footrot). Long term disease with footrot [3,5] and poor foot trimming [4] can alter foot integrity.

A diagnosis of footrot can be made using culture or PCR from swabs taken from the hoof horn junction [6]. However, these laboratory methods are not completely reliable. *D. nodosus* requires complex media for culture with strict anaerobic conditions [6], and while 16S rRNA PCR is more rapid and sensitive than culture it is still far from 100% sensitive [7]. As a consequence, diagnosis using visual observation of the foot without further laboratory tests is commonly used by researchers and clinicians once *D. nodosus* is endemic in a flock. Visual diagnosis may include a system to score the severity of the footrot lesion. A commonly used system to score footrot is an Australian system with five ordinal scores [8] (Table 1). In the UK, in addition to scoring footrot, a 4 point ordinal scoring method to score foot integrity has been used [4]. These scoring systems have been used by researchers [5,9,10] to study the epidemiology, pathogenesis, treatment, control and economic losses attributable to footrot. However, the between and within observer reliability of a scoring method for foot integrity has not been formally tested. One study [11] investigated agreement of a footrot scoring system between two trained observers and reported a high level of agreement, but the study had 85% of lesion score 0 (no lession) out of 100 sheep. The study provided no information on when the observers disagreed or where (i.e. which scores) the disagreement lay.

**Table 1:** Footrot scoring scale from Egerton and Roberts (1971)

Score	Description
score 0	Normal foot
score 1	Limited mild interdigital dermatitis
score 2	More extensive interdigital dermatitis
score 3	Severe interdigital dermatitis and under-running of the horn of the heel and sole
score 4	As 3 but with the under-running extended to the walls of the hoof

The reliability of a numeric scoring system is the generalizability (based on generalizability theory) of the results across scoring situations and judges [12]. To evaluate this, reproducibility (as a measure of between observer variability) and repeatability (the measure of within observer variability) are estimated [13]. In both the medical and veterinary fields, an ordinal score is often used to evaluate the severity of a disease [14]. The observer agreement for such ordinal data is commonly provided by a single measure of agreement e.g. weighted kappa coefficients [15] or Kendall’s coefficient of concordance [16]. These do not provide information on components of disagreement such as observer bias (i.e. tendency for observers to give higher of lower rating than others) or differences in thresholds and therefore category widths for the ordinal scale. There is one study by Thomsen et al. [17] that tested whether the category widths used by observers for an ordinal scale were equidistant by calculating a polychoric correlation. But this approach only compared two observers and did not provide an estimate for observer bias.

Modeling techniques have been described to evaluate observer agreement for ordinal scores. These include log linear models [18], association models [19] and latent trait and latent class models [20-22]. Both log linear and association models have been designed to compare only two observers and there are issues with interpretation of relative magnitude of some of the parameters used [14]. Latent trait and latent class models have been designed for multiple observers and have been used in the medical field [21,22] to quantify agreement with multiple observers. These models explore agreement by testing whether there is observer bias and give a visual representation of the observers’ perceived impressions of the scores on a continuum, thus indicating the threshold and width of score categories, for example, for a 0 to 3 category scale, the first threshold is the point from which an observer applies score 1 and below that would be score 0, the second threshold the point from which an observer applies score 2 and so on. To our knowledge such modeling approaches have not been used to evaluate observer agreement for ordinal categories in the veterinary field. In the current paper, observer agreement of scoring systems for footrot (using photographs and videos) and foot integrity (using *post mortem* feet) in sheep is evaluated and explored using two approaches, weighted kappa and located latent class modeling.

## Methods

### Scoring systems

We used a five point ordinal the scoring system (0–4) proposed by Egerton and Roberts [8] (Table 1) to score photographs and videos of footrot and a four point ordinal scale to score foot integrity proposed by Kaler et al. [5] (Table 2).

**Table 2:** Foot integrity scoring scale (sole and wall are scored separately)

Score	Description of sole/wall of digit
0	Undamaged sole/wall area with a perfect shape
1	Mildly damaged/misshapen sole/wall area of the digit (<=25%)
2	Moderately damaged/misshapen sole/wall area of the digit (>25% and 75%)
3	Severely damaged/misshapen sole/wall area of the digit (>75%)

### Study design

#### Videos and photographs of footrot lesions

**Figure 1:**
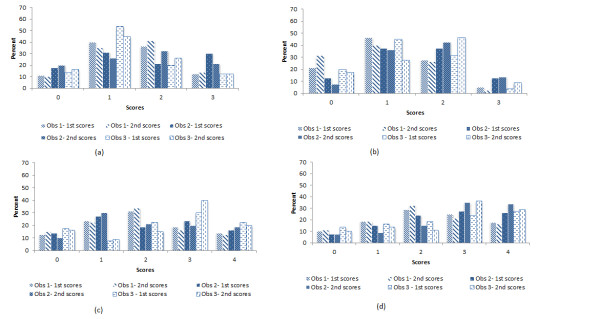
First and second scores of sole integrity (a), wall integrity (b), footrot using photographs (c) and footrot from videos (d) by observers.

120 video clips of sheep feet with footrot scores ranging from 0–4 (Table 1) were made on farms with informed consent from farmers in the UK, Sardinia and India. Videos were recorded using a JVC (GR-D21) or a Sony camcorder (HDR-SR10E) and edited using Movie Maker (Windows 2007). Eighty videos clips were selected that included the range of scores (Figure 1). Eighty photographs were made from snapshots of footrot lesions from the video clips. The identification number for the video and picture of the same footrot lesion were different.

#### Feet with different foot integrities

A total of 500 sheep feet were collected from an abattoir over two weeks. Feet were washed and then stored at −20°C. A total of 80 feet with the range of integrity scores were selected. Feet were removed from the freezer and left overnight to thaw before being scored.

#### Data collection

Three observers with some previous experience of scoring feet were selected. Observers were trained for one hour before they were tested. They scored footrot (Table 1) from the 80 photographs and videos which were given to them on two cds. Each photograph was shown for five seconds with a four second lag between each photograph and each video lasted 20–25 sec with four seconds lag between each video clip. On day 1, the photographs were scored twice (re-ordered the second time) by observers with a gap of 2 hours between the two scorings sessions. On day 2, observers scored videos twice with a gap of 2 hours between the scoring sessions.

Observers scored the integrity of the soles and walls (Table 2) of the 80 digits in a preparation room. They had 30 seconds to examine and score each digit. They repeated the scoring after an interval of 2 hours. Observers did not discuss their observations with each other.

#### Statistical analysis

Data were entered in Microsoft excel (Microsoft Office 2007) and analysed using STATA 10.0 (StataCorp, USA) and LLCA [21].

#### Weighted kappa

The weighted kappa (Kw) was calculated within observers and between pairs of observers. The Kw values were interpreted according to Landis and Koch [14], 0 = poor, 0.01–0.20 = slight, 0.21–0.40 = fair, 0.41–0.60 = moderate, 0.61–0.80 = substantial and 0.81–1 = almost perfect.

#### Located latent class analysis

To investigate components of disagreement a located latent class analysis as described by Uebersax [21] was performed. The located latent class model works on the theoretical principle that there is a unidimensional continuum of a latent trait *θ* that is a basis for ratings which is assumed to range from -∞ to ∞. The latent trait in the current study was the ordinal scoring scale. Different ordinal categories (i) of the scoring scale were represented as latent classes (c) which presented themselves as discrete locations on this continuum and were assumed to correspond to the true latent trait level ($\beta_{c}$). Each observer (r) had i-1 ordered thresholds ($t_{\mathrm{ir}}$) on this continuum which was the observer’s perceived impression (apparent trait level) of an ordinal category. For the 0 to 3 category scale, there are three thresholds 0–1, 1–2 and 2–3 and similarly for the 0 – 4 ordinal scale there are four thresholds. Due to measurement error α (which is assumed to be normally distributed), the apparent trait levels of latent class c varied from $\beta_{c}$. The model took the form:

(1) $$\varphi_{\mathrm{cr}}(\theta )=\{1+\exp{\left[-1.7\alpha_{r}(t_{\mathrm{ir}}-\beta_{c}]\right\}}^{-1}$$

where $\varphi_{\mathrm{cr}}(\theta )$ is the logistic cumulative density function of the apparent trait level of latent class *c* for observer *r*. The model was run in LLCA FORTAN [21] and maximum likelihood was used to quantify observer bias (differences between observers’ mean thresholds) and category widths (distance between individual thresholds $t_{\mathrm{ir}}$ for categories). Two sub-models were created by adding constraints to the basic model (Equation 1) to test whether there was significant observer bias and significant differences in ordinal category widths for between and within observers. Sub-models were defined:

(2) $$t_{\mathrm{ir}}=\Delta_{r}+\delta_{\mathrm{ir}}$$

where $\Delta_{r}$ was the mean threshold of observer r and $\delta_{\mathrm{ir}}$ was the deviation of threshold $t_{\mathrm{ir}}$ from $\Delta_{r}$. In the first sub-model (simple bias model), to test observer difference in category widths, a constraint was applied by restricting $\delta_{i1}=\cdots \delta_{\mathrm{ir}}$ so that category widths were the same across the observers and observers differed by an overall bias. This was nested in the basic model (Eq1) and compared. For the second sub-model (identical threshold model) $\Delta_{1}=\cdots \Delta_{r}$, (equal bias across observers) was restricted and this model was nested in the simple bias model and compared. A likelihood ratio chi-square test was used to compare both sub-models; p-values <0.05 were considered significant. Estimated threshold locations with bias parameters and confidence intervals were compared. Further details of the methodology of LLCA are presented in Ubersax [21,22]. For between observer agreement, observers 1^st^ scores were used.

The verification of the model assumption of unidimensional latent trait was done by confirming a single high Eigen value of polychoric correlation between pair of observers [22].

## Results

The distributions of scores between and within observers for photographs and videos of footrot lesions and foot integrity scoring scales are presented in Figure 1.

### Footrot scoring scale

a) Weighted kappaThe weighted kappa values between observer pairs for footrot ranged from moderate to substantial; 0.57 to 0.65 for photographs and 0.65 to 0.73 for videos. The within observer weighted kappa values were higher and ranged from substantial to almost perfect: 0.78 to 0.91 for pictures and 0.77 to 0.89 for videos (Table 3).

**Table 3:** Between and within observer weighted kappa (Kw) and 95% confidence intervals for footrot and foot integrity scores

	Weighted kappa (95% CI)			
	Footrot scores		Foot integrity scores	
	Pictures	Videos	Soles	Walls
Between Observers				
Observer 1–2	0.58 (0.41-0.74)	0.68 (0.52-80)	0.68 (0.58-0.77)	0.58 (0.45-0.71)
Observer 1–3	0.67 (0.49-0.80)	0.65 (0.44-0.78)	0.70 (0.58-0.71)	0.70 (0.58-0.79)
Observer 2–3	0.65 (0.46-0.75)	0.73 (0.61-0.83)	0.67 (0.57-0.77)	0.68 (0.55-0.75)
Within Observer				
Observer 1	0.90 (0.70-0.98)	0.89 (0.80-0.93)	0.90 (0.83-0.95)	0.84 (0.76-0.90)
Observer 2	0.78 (0.67-0.75)	0.77 (0.64-0.86)	0.86 (0.78-0.92)	0.82 (0.72-0.89)
Observer 3	0.91 (0.71-0.97)	0.85 (0.75-0.92)	0.83 (0.74-0.89)	0.73 (0.64-0.83)

**Figure 2:**
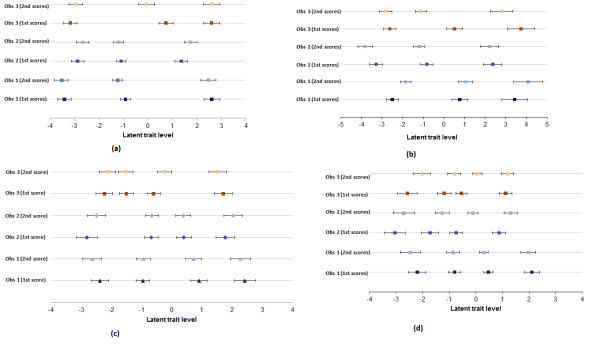
**Estimated threshold locations (square) with 95% confidence intervals of scores of sole (a) and wall (b) integrity, footrot using photographs (c) and videos (d) for observer’s first and second scoring sessions.** The first location is the threshold above which observers apply score 1 (below that threshold score 0 is given) and so on for successive scores.

b) Located latent class modelThe observer thresholds for lesion scores using photographs and videos at their first and second scoring sessions are presented in Figure 2. For photographs, all the observers had similar threshold locations for score 0 and score 4 and for videos, the threshold location for score 0 was similar between observers but varied for other scores.

### Category widths

The category widths (i.e. the distance between adjacent thresholds) for the lesion scale varied significantly between observers with both photographs and videos. Observer 3 had significantly narrower widths for scores 1 and 2 for photographs and score 2 for videos compared with the other two observers (Figure 2). Category widths for the scores did not vary significantly within observers for photographs or videos.

### Observer bias

There was evidence of significant between observer bias when scoring lesions from photographs; observer 3 had a significantly lower mean threshold (−0.437) compared with observers 1 (0.318) and 2 (0.069) (Table 4). Similarly, there was significant bias between observers in their scores for videos; observer 1 (0.566) had a higher mean threshold compared with the other two observers (−0.676 (obs 2);−0.333 (obs 3)) with observer 3 having a comparatively higher threshold than observer 2. There was no evidence of bias within observers when scoring photographs. However, when scoring videos, observers 2 (0.676 vs−0.083) and 3 (0.122 vs −0.333) had significantly higher mean thresholds at the second scoring session compared with the first session (Table 4).

**Table 4:** Estimates of observer mean thresholds (standard errors) as an index of observer bias for footrot and foot integrity scores

	Footrot scores		Foot integrity scores	
Observer	Pictures	Videos	Soles	Walls
Observer 1- 1st score	0.318 (0.124)	0.566 (0.112)	−0.035 (0.140)	0.541 (0.169)
Observer 1 - 2nd score	0.214 (0.124)	0.404 (0.123)	−0.251 (0.136)	1.086 (0.164)
Observer 2- 1st score	0.069 (0.128)	−0.676 (0.130)	−0.436 (0.139)	−0.607 (0.156)
Observer 2 - 2nd score	0.181 (0.127)	−0.083 (0.131)	−0.241 (0.139)	−0.963 (0.161)
Observer 3- 1st score	−0.437 (0.128)	−0.333 (0.135)	0.511 (0.151)	0.439 (0.165)
Observer 3 - 2nd score	−0.346 (0.127)	0.122 (0.132)	0.401 (0.145)	−0.497 (0.164)

### Foot integrity scale

a) Weighted kappaBetween observer weighted kappa values ranged from moderate to substantial; 0.67 to 0.70 for soles and 0.58 to 0.70 for walls. Within observer weighted kappa values were higher than between observer with substantial to almost perfect agreement and ranged between 0.83 to 0.90 for soles and 0.73 to 0.84 for walls (Table 3).

b) Located latent class modelThe observer threshold locations for foot integrity scores of soles and walls at the first and second scoring sessions are presented in Figure 2.

### Category widths

There were significant differences in the category widths of scores between observers for soles and walls. Scoring soles, observer 2 had a wide category for score 3; observer 3 had a wide score 1 category and a narrow score 2 category compared with the other two observers. Scoring walls, observer 2 had smaller category width for score 0 compared with the other two observers. Sole category widths did not differ significantly within observers, however, within observer 3 there were different category widths for the middle scores for wall integrity (Figure 2).

### Observer bias

There was significant bias between observers for scoring foot integrity of soles and walls (Table 4). Observer 3, had a significantly higher (0.511) mean threshold for scoring soles compared with observers 1 (−0.035) and 2 (−0.436) with observer 2 having higher mean threshold than observer 1. Observer 2 had a lower (−0.607) mean threshold for scoring walls compared with the other two observers (0.541 (obs 1); 0.439 (obs 1)).

There was no bias within observers for scores of sole integrity; however, there was significant bias within all observers for scores of wall integrity. Observers 2 and 3 had a lower mean threshold value and observer 1 had a higher mean threshold value at their second scoring session compared with their first session (Table 4).

## Discussion

This paper explores components of disagreement between and within observer scoring for two visual ordinal scales. For both photographs and videos of footrot and foot integrity, the within observer agreement was higher than the between observer agreement suggesting that these scoring systems are most reliable when used by the same person. This is evident from both the weighted kappa values (showing moderate to substantial agreement between observers and substantial – almost perfect agreement within observers) and the LLCA (Figure 2) where the threshold locations for ordinal scores were very different between observers but less so within observers.

The high within observer agreement could have occurred because there was a gap of only two hours between the two scoring sessions and observers remembered their scores which reduced the within observer variability, however, there were 80 items (feet/photographs/videos) to score and they were re-ordered between sessions so this seems unlikely. Another possible explanation for high within observer reliability is that the within observer agreement is less likely to be affected by some additional sources of variation that exist between observers e.g. different experiences and different inherent score definition among different observers which reduce reliability. These sources of variation could have resulted in differences in the score thresholds and bias between observers and the poor between observer reliability as seen in this study. Knowledge of where the disagreement lies between observers by getting information on their thresholds for each score is useful to identify particular scores where observers have most disagreement. For example, scoring photographs and videos of footrot this was for scores 1 and 2 (Figure 2). Visual representation of thresholds, and where a discrepancy lay could help train observers and reduce between observer differences and so improve reliability. It could also be used to make improvements in particular score definitions for an existing scoring system and also could be used during development and training of a new scoring system.

Unlike footrot where there are more clear signs that differentiate a diseased foot from normal, there was more within observer subjectivity in categorising the wall of the foot as mildly misshapen or normal. The overall observer agreement for walls was lower than that for soles this could be because the smaller surface area and relatively flat anatomical presentation of the soles, of the foot, in comparison to walls, makes scoring easier and more consistent.

The observers’ reproducibility and repeatability for scoring video clips and feet (integrity) were both lower than scoring from photographs. There was a difference in the length of time for which feet, videos and photographs were shown which might account for this difference but it might also be that a still 2D image of the foot was easier to score consistently than all-around video footage or a 3D digit where observers had several views and so could make several interpretations. In reality, it is quite possible that feet and videos clips although less reliably scored are more similar to real life than a photograph.

We considered the use of live sheep for this study, however, the possible change in footrot lesions over time [23], (even within hours the foot can change in highly conducive environment) and the difficulty in restraining live sheep to allow controlled observation of the feet for a specified time period would have introduced unnecessary error into the study. In addition, it is unlikely we would have been able to represent the whole range of scores in sufficient number in a flock of sheep at one point in time (as can be seen in the paper by [11]) and to run the study over time would again have introduced error. For these same reasons other studies have used videos or photographs to test observer agreement in scoring locomotion or injuries in different species such as horses [24,25] cows [26], sheep [27] and dogs [28]. In addition, such an approach is a refinement on the use of animals in research; all the sheep that were videoed in this study were being examined as part of normal farming practice and those with lesions were treated immediately. A future study with 2 observers simultaneously scoring footrot lesions on live sheep to test between observer bias when observing live sheep would be useful, but carries the provisos of numbers of sheep with each score as above [11].

There is a growing literature on the drawbacks of using kappa values to assess observer agreement. Weighted kappa values influence the prevalence of each score, the marginal distributions of scores given by observers [29] and the chosen weights in an ordinal scale [16]. As also evident from the current study, Weighted kappa values provide no information on sources and types of disagreement [15]. In contrast, the located latent class analysis presented here is a very useful method to investigate agreement in ordinal scales and gain a visual insight into the various sources of disagreement. It could be particularly useful when developing and piloting a scoring system to identify sources of disagreement and make improvements to the score definitions.

## Conclusions

Located latent class analysis is a useful technique to unravel sources of disagreement between observers. In the current study, although both the footrot and foot integrity scoring scales had moderate to high between observer agreement there was observer bias and differences in category widths between observers. The difference in category widths between observers occurred mainly in the middle categories (score 1 and 2) for footrot scores when scored using photographs and videos and for all categories for foot integrity scores. This indicates that improvements in the scoring systems are required. Currently, given that the within observer agreement was almost perfect and category widths were consistent these scales are most reliable when scored by the same person.

## Competing interests

Authors declare that they have no competing interests.

## Authors’ contributions

JK participated in the design of the study, data collection, performed the statistical modeling and drafted the manuscript. AF participated in the study design, data collection, performed weighted kappa analysis and contributed to a first draft of the manuscript. LEG participated in the study design, discussion on analysis and in the preparation of the final manuscript. SAM contributed to the statistical programming. All authors read and approved the final manuscript.
